# Supplementary material for: Iterative improvement in the automatic modular design of robot swarms
Source: PeerJ Comput Sci. 2020 Dec 7;6:e322. doi: 10.7717/peerj-cs.322 (PMC7924708; doi:10.7717/peerj-cs.322)
Supplement: Supplemental Information 3 [file peerj-cs-06-322-s003.zip › argos3/doc/api/standalone/a00435_source.html]

ARGoS: plugins/robots/foot-bot/control\_interface/ci\_footbot\_base\_ground\_sensor.h Source File


- Main Page
- Related Pages
- Namespaces
- Classes
- Files

- File List
- File Members

# plugins/robots/foot-bot/control\_interface/ci\_footbot\_base\_ground\_sensor.h

Go to the documentation of this file.

```
00001 
00032 #ifndef CCI_FOOTBOT_BASE_GROUND_SENSOR_H
00033 #define CCI_FOOTBOT_BASE_GROUND_SENSOR_H
00034 
00035 namespace argos {
00036    class CCI_FootBotBaseGroundSensor;
00037 }
00038 
00039 #include <argos3/core/control_interface/ci_sensor.h>
00040 #include <argos3/core/utility/math/vector2.h>
00041 #include <vector>
00042 
00043 namespace argos {
00044 
00045    class CCI_FootBotBaseGroundSensor : virtual public CCI_Sensor {
00046 
00047    public:
00048 
00049       struct SReading {
00050          Real Value;
00051          CVector2 Offset;
00052          
00053          SReading() :
00054             Value(0.0f) {}
00055          
00056          SReading(Real f_value,
00057                   const CVector2& c_offset) :
00058             Value(f_value),
00059             Offset(c_offset) {}
00060       };
00061 
00062       typedef std::vector<SReading> TReadings;
00063 
00064    public:
00065 
00066       CCI_FootBotBaseGroundSensor();
00067 
00068       virtual ~CCI_FootBotBaseGroundSensor() {}
00069       
00070       const TReadings& GetReadings() const;
00071       
00072 #ifdef ARGOS_WITH_LUA
00073       virtual void CreateLuaState(lua_State* pt_lua_state);
00074 
00075       virtual void ReadingsToLuaState(lua_State* pt_lua_state);
00076 #endif
00077 
00078    protected:
00079 
00080       TReadings m_tReadings;
00081 
00082    };
00083 
00084    std::ostream& operator<<(std::ostream& c_os,
00085                             const CCI_FootBotBaseGroundSensor::SReading& s_reading);
00086 
00087    std::ostream& operator<<(std::ostream& c_os,
00088                             const CCI_FootBotBaseGroundSensor::TReadings& t_readings);
00089 
00090 }
00091 
00092 #endif
```

---

Generated on 10 Jul 2018 for ARGoS by 
 1.6.1
